# Workload Determines Workplace Stress among Health Professionals Working in Felege-Hiwot Referral Hospital, Bahir Dar, Northwest Ethiopia

**DOI:** 10.1155/2018/6286010

**Published:** 2018-11-26

**Authors:** Minyichil Birhanu, Berhane Gebrekidan, Getasew Tesefa, Minale Tareke

**Affiliations:** ^1^Bahir Dar University, College of Medicine and Health Science, Bahir Bar, Ethiopia; ^2^Addis Ababa University, College of Health Sciences, School of Allied Health Sciences, Department of Nursing and Midwifery, Addis Ababa, Ethiopia

## Abstract

**Background:**

Workplace stress occurs in all professionals but, in particular, health-care professionals are highly prone to workplace stress. Health-care professionals comprise an important group that can be impacted by workplace stress because of their unique work environment. The study was done to determine the level of workplace stress and its associated factors among health-care professionals in Felege-Hiwot Referral Hospital, Bahir Dar, Northwest Ethiopia.

**Methods:**

An institutional-based cross-sectional study was conducted among randomly selected health-care professionals in Felege-Hiwot Referral Hospital. Data were collected using a self-administered structured questionnaire by trained data collectors and the supervisor. The collected data were entered into EPI-info version 7 and exported to SPSS version 20 for analysis. Logistic regression was employed to assess the associations between dependent and explanatory variables.

**Results:**

This study revealed that prevalence of workplace stress was 68.2%. Health professionals who work 50 hours and more per week and in night shift on sometimes base were more likely to develop workplace stress.

**Conclusion:**

The level of workplace stress among health professionals was found to be high. This was due to long working hours and working in night shift. Identifying the source of workplace stress among health professionals should be a great concern for health service managers and other stakeholders.

## 1. Background

Stress is a multidimensional concept which is originally derived from the Latin word, “stringere” which refers to draw tight, to describe hardships and/or affliction [1]. It often occurs when individuals' physical and emotion do not match or cannot handle their job demands, constraints, and/or opportunities. “Stress may be established on two major types of stress: eustress (good stress) and distress.” Individuals who experience eustress will be able to meet job demands, and this may help them to increase positive work life (e.g., satisfaction and positive moral values) [2].

On the contrary, “individuals who experience distress will not be able to fulfill job demands and this may motivate them to decrease quality of work life (e.g., dissatisfaction and negative moral values)” [3]. Consequently, it may decrease the ability of employees to control and manage physiological and psychological stresses, such as disturb their self-regulatory bodies, and they cannot meet their duties and responsibilities as a member of an organization [4]. Stress can be generally defined as undue, inappropriate, or exaggerated response to a situation [5]. Stress, particularly work stress, is said to cause fatigue, depression, and tension on people and employees in all types of businesses and industries [6].

Globally, work-related stress is estimated to affect one in three employees [7]. Workplace stress occurs in all professions, and in particular, health-care professionals comprise an important group that can be affected by workplace stress because of the nature of their work environment [8]. In health care, employee workplace stress can have a negative impact on the quality of patient care [9], and significant effect on the occurrence of health problems leading to change the current working place and job, quit the profession, and interrupt relationship with coworkers [10]. In general, different studies confirmed that work stress may lead to negative financial outcomes. Globally, the cost of work-related stress is estimated to be approximately $5.4 billion each year, second to low back pain which is the most frequent occupational health problem [7].

In particular, stress in the nursing profession has been a major worldwide problem. A study among a large sample of Swedish nurses revealed that more than 80% of the nurses reported high job strain [11]. A study among personnel of a UK health authority reported that nurses were under the greatest pressure among all health-care personnel [12]. The study estimated that almost 10% of the gross national product in European countries is lost because of stress-related absenteeism and turnover [13].

The magnitude of the problem was further emphasized in the report of the American Foundation for Suicide Prevention which claimed that, on the average, death by suicide is about 70% more likely among physicians than among other professionals and 250–400% higher among female doctors. The major cause is stress and depression thereof [14]. It has been shown that health workers are highly prone to stress at work and experience more negative outcomes of stress than other professionals. Work stress in physicians will cause negative outcomes like adverse psychological well-being, job burnout, and significantly larger number of suicide attempts, alcohol dependency, and other psychosocial problems [15]. Stress may also harm professional effectiveness: it decreases attention [16], reduces concentration [17], impinges on decision-making skills [18], and reduces providers' abilities to establish strong relationships with patients [19]. Stress also may lead to increased burnout and is defined as a syndrome of depersonalization, emotional exhaustion, and a sense of low personal accomplishment [20].

According to the International Labour Organization, almost 10% of workplace accidents are related to stress seeing that the ability to effectively manage stress can help maintain organization harmony [21]. In the hospital, most of the employee stress is caused by work overload, repetitive duties, inadequate resources, physical environment (lighting, space, temperature, and disruption), psychological working environment (verbal abuse and inappropriate behaviours), working long hours, management issues, inadequate allocation of work, new technology, and others [22]. The study conducted to identify sources of stress in health-care personnel of each category showed that the prime sources of stress were underpayment, excessive workload, inadequate staff, and being involved in the emotional distress of patients [23].

Assessing workplace-related stress and its contributing factors among health professionals are important for the health professionals, patients, and organizations. Therefore, the aim of this study was to determine the level and factors of workplace stress among health professionals in Felege-Hiwot Referral Hospital, Bahir Dar, Northwest Ethiopia.

## 2. Methods

An institutional-based cross-sectional study was conducted among health-care professionals in Felege-Hiwot Referral Hospital from March 1 to 20, 2015. Felege-Hiwot Referral Hospital is found in the capital city of Amhara Regional State, Bahir Dar, which is 565 km far away from the capital city of Ethiopia, Addis Ababa. It was established in 1963 as the district hospital, and it was upgraded to referral hospital in 1994. The hospital has Surgery, Medical, Pediatrics, Obstetrics and Gynecology, Psychiatry, Dental, and Orthopedics units with both outpatient and inpatient departments and follow-up departments. The hospital has a total of 415 health professionals. Sample size was determined by single population proportion formula, and it was 208.

### 2.1. Sampling Procedure

All health professionals found in the hospital were stratified based on their profession (job). Stratified sampling technique was used to select the study units in each stratum. Based on the number of professionals found in each stratum, proportional allocation of the total sample size was carried out to attain the required sample size. Finally, the determined sample size for each stratum was recruited using simple random sampling technique.

### 2.2. Data Collection

Data were collected using a self-administered questionnaire. The questionnaire consisted of sociodemographic information, working environment, and common outcomes of stress, causes of stress, and stress-level measuring tool. The stress causes-related questionnaire contained 39 statements which were developed in a way that allows respondents to grade their responses on a five-point scale: strongly disagree = 1, disagree = 2, do not know = 3, agree = 4, and strongly agree = 5 [10]. The level of stress was measured using the Perceived Stress Scale (PSS) which was used by the World Health Organization (WHO) to examine personal stress. The questions were contextualized to workplace making participants to respond by correlating it to stress at workplace rather than stress at home. The scale was scored into 5 points like 0: never, 1: almost never, 2: sometimes, 3: fairly often, and 4: very often. Then, the scores for questions 4, 5, 7, and 8 were reversed. Then, scores for each item were added to get a total score. Individual scores on the PSS can range from 0 to 40 with higher scores indicating higher perceived stress. Scores ranging from 0 to 13 were considered no stress while score 14 and above was considered as stress [24].

### 2.3. Data Quality Assurance

A pre-test was conducted using 5% of the sample among health professionals in the Bahir Dar health centre to assess instrument simplicity, flow, and consistency. Some modifications were made based on the result from the pre-test. Data were collected by one-day trained data collectors and a supervisor. Data completeness and consistency were checked by the supervisor and investigators.

### 2.4. Data Processing and Analysis

Data were entered and cleaned using Epi-Info version 7 and exported to Statistical Package for Social Science (SPSS) version 20 for analysis. Descriptive analysis was used to present results. Binary logistic regression was used to assess associations between dependent and independent variables. The degree of associations was interpreted using odds ratio (OR) and 95% confidence interval (CI) at 0.05 *p* value.

### 2.5. Ethical Consideration

Ethical clearance was obtained from Addis Ababa University, College of Health Sciences; a research review committee before data collection. Support letter was obtained from Felege-Hiwot Referral Hospital officials and concerned stakeholder. Oral consent was obtained from each study participants during data collection. The right was given to study participants to refuse, stop, or withdraw from the interview at any time. Confidentiality was maintained throughout the study.

## 3. Result

### 3.1. Sociodemographic Characteristics

Of the 208 sample health professionals, data were collected from 198 health professionals with a response rate of 95%. A majority of the participants (55.1%) were males. The ages of the respondents ranged from 23 to 59 years with a mean age of 31.33 ± 7 years, and 111 (56.1%) were married. Most respondents (89 (44.9%)) have Bachelor of Science degree (Table 1).

### 3.2. Working Units

**Figure 1:**
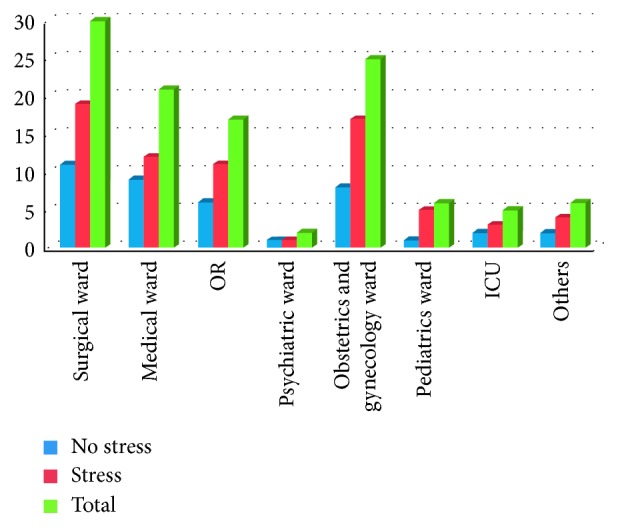
Distribution of health professionals by their inpatient working unit and stress level in Felege-Hiwot Referral Hospital, Bahir Dar, Northwest Ethiopia, 2015.

**Figure 2:**
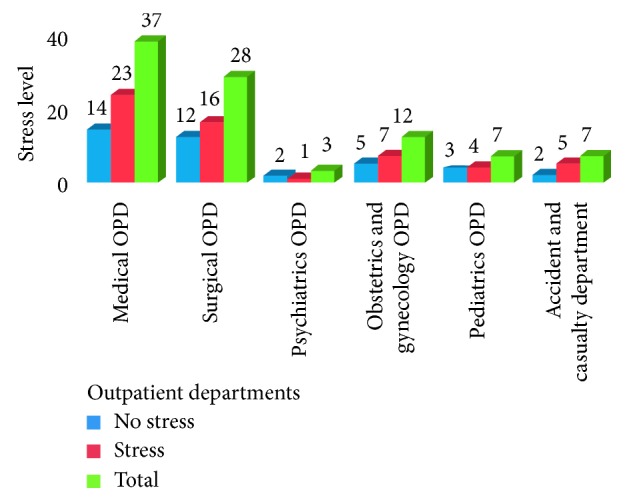
Distribution of health professionals by their outpatient department working unit and stress level in Felege-Hiwot Referral Hospital, Bahir Dar, Northwest Ethiopia, 2015.

Among respondents who work in inpatient departments, the majority (30 (26.78%)) were from the surgical ward (Figure 1); and from study participants who work in the outpatient department, most (39.6%) were from the medical OPD (Figure 2).

### 3.3. Job Characteristics

Most (85 (42.9%)) health professionals have high workload (>50 hr/week). A majority (79.3%) of respondents worked in night shift sometimes. One hundred eight (54.5%) health professionals work on night and weekend call duties in addition to their daily work on sometimes base. One hundred fifty-six (78.8%) health professionals believed that there is no adequate staff to do the job properly. The result showed that 164 (82.2%) of respondents did not spend enough time with their family, and 125 (63.1%) of respondents had close friends or family in their workplace. A majority (152 (76.8%)) of health professionals did not get extracurricular activities from their hospital administration that would decrease the stress level in their workplace and did not spend enough time with their family 162 (82.2%) (Table 2).

### 3.4. Stress Level

The PSS score for the study participants ranged from 3 to 28 with a mean of 15.97 ± 5.25. Using the PSS score of 14 and above, the prevalence of workplace stress in this study was 68.2% (95% CI: 62.8–73.2).

**Figure 3:**
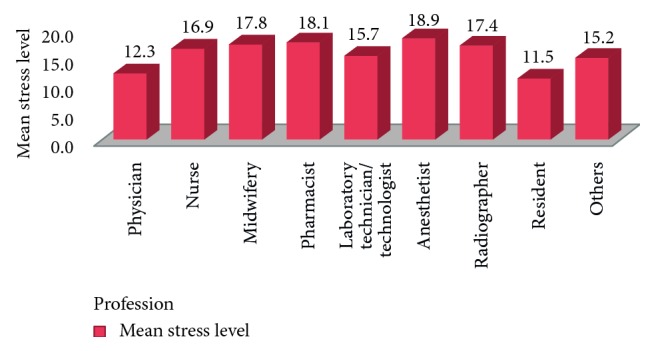
Comparison of workplace stress level among different professional groups in Felege-Hiwot Referral Hospital, Bahir Dar, Northwest Ethiopia, 2015.

The result revealed that the mean scores of the workplace stress level among anaesthetists, pharmacists, midwiferies, radiographers, nurses, laboratory technicians/technologists, psychiatrists/sanitarian/health educators, physicians, and residents were 18.9, 18.1, 17.8, 17.4, 16.9, 15.7, 15.2, 12.3, and 11.5, respectively (Figure 3**)**.

### 3.5. Anticipated Outcome of Workplace Stress

**Figure 4:**
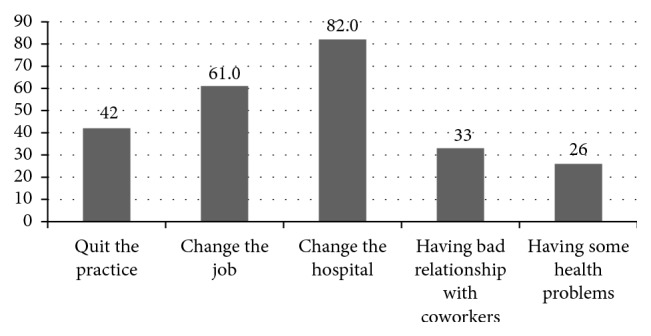
Anticipated results of workplace stress among health professionals in Felege-Hiwot Referral Hospital, Bahir Dar, Northwest Ethiopia, 2015.

Nearly one-third of health professionals planned to change the current hospital, change the job (61 (25%)), and plan to quit the practice (42 (17%)); 33 (14%) had bad relationship with coworkers, and 26 (14%) developed some health problems as a result of workplace stress (Figure 4).

### 3.6. Factors Associated with Workplace Stress among Health Professionals

All independent variables were checked for association with the dependent variable by binary logistic regression. On bivariate analysis, the factors *p* value less than 0.2 were age, educational level, job, monthly income, workload, working in night shift, working in weekend, demand conflict on time, spend enough time, and knowing whom to approach where there is stress.

In order to control the possible confounder's effect, *p* value of less than 0.2 in bivariate analysis was included in the multivariate analysis. The multivariate analysis indicated that workload and working in night shift were significantly associated with stress with a *p* value less than 0.05.

The result revealed that health professionals who work >50 hr/week were ten times more stressed than those who work 40 hr/week (AOR: 9.630, 95% CI: 2.410–38.460). Respondents who work 41–50 hours per week were 6 times more stressed than those who work 40 hours per week (AOR: 5.98, 95% CI: 9.935–35.997). Study participants who work in night shifts on sometimes base were highly stressed than those who never work in night shifts (AOR: 3.6, 95% CI: 1.009–13.286) (Table 3).

## 4. Discussion

In this study, the prevalence of workplace stress was 68.2% which is in line with the study in Saudi Arabia (66.2%) [25] and India (73.5%) [26]. However, in the present study, the prevalence was higher than results reported from Dutch (55%) [27], Belgium (40.4%) [28], Iran (34.9%) [29], and other parts of Ethiopia (37.8%) [7]). The possible reasons for the difference might be due to differences in tools used, study participants (previous studies included only one professional group), and relative small sample size in the current study.

Similar to the finding from the study done in Saudi [10], there was no statistically significant difference between sex and marital status and workplace stress in the current study. However, reports from Lodz, Poland [30], and other parts of Ethiopia (AOR: 2.47, 95% CI: 1.28, 4.77) [7]) pointed that female health professionals were more likely to have stress than males. Furthermore, the study in other parts of Ethiopia showed that widowed and divorced nurses were 10 times more likely to experience workplace stress than married nurses (AOR: 10.11, 95% CI: 4.56–15.17) [7]. These differences might be due to difference in study participants showing that these two studies included only nurses, but the current study included all health professionals.

In this study, health professionals' educational level did not significantly influence the level of workplace stress, which is consistent with the study in Saudi Arabia [25], Iceland [31], and other parts of Ethiopia [7]. In contrast, the result from Saudi brought out that the work-stress level was low among those holding postgraduate degrees [10]. Although the difference was not statistically significant, the present study reported that workplace stress was higher among anaesthetists (mean stress level 18.9) and lower among residents (mean stress level 11.5). Nevertheless, work stress was higher among doctors (stress level = 4.04) and lower among hospital administrators (stress level = 3.69) in Saudi Arabia [10]. The reasons for the difference might be difference in sample size and tools used.

The current study revealed that there was no significant association between monthly income and stress level among health professionals. But, the study in Germany and Austria among physicians, radiographers, nurses, and medical physicists working in radiotherapy showed that the greatest stress factors for nurses were “underpayment” [32]. This difference might be due to differences in tools used. The study in Saudi Arabia showed that those who work more than 50 hours per week were more stressed (79.4%; *p*=0.001) [25]. Similarly, this study disclosed that those who work more than 50 hours per week were more stressed than those who work 40 hours per week (AOR: 9.6, 95% CI: 2.410–38.46); and those who work 40–50 hours per week were six times more stressed than those who work 40 hours per week (AOR: 5.98, 95% CI: 9.935–35.997). The current study showed that those who work in night shifts on sometimes base were more stressed than who never work in night shifts (AOR: 3.66, 95% CI: 1.009–13.286). It coincides with the study in Saudi Arabia reported that those who always worked in night shifts were 84.0% more stressed (*p* < 0.001) compared to those who never or sometimes worked [25] which is supported by the findings from Germany and Austria indicating that the main source of workplace distress was working in night shifts [32].

In the current study, frequently stated sources of workplace stress by physicians were “Job-requirement is more than my ability” (mean score 3.72), “work shifts are changing frequently” (mean score 3.72), “working with opposite sex” (mean score 3.69), “feeling isolated” (mean score 3.66), and “hospital objectives do not match mine” (mean score 3.66). But, the study in Germany and Austria showed that the four greatest sources of a physician's job stress were “too much office work” (mean score 3.4), “time pressure” (mean score 3.36), “ill-defined responsibilities” (mean score 3.13), and “breaking off the conversation with the patient” (mean score 3.10). The variation with the current study might be due to differences in setting and sample size.

Nurses frequently stated “working with opposite sex” (mean score 3.35), “feeling isolated” (mean score 3.31), “lack of stability at home” (mean score 3.30), and “supervising the work of other people” (mean score 3.29) as the source of workplace stress. However, the study in Germany and Austria showed that “permanent ringing of telephone” (mean score 3.53), “against the conviction patients were kept alive by all means” (mean score 3.22), “underpayment” (mean score 3.21), and “time pressure” (mean score 3.11) were sources of workplace stress for nurses. The variation with the current study might be due to differences in setting and sample size.

For radiographers, frequently stated sources of workplace stress were “working with opposite sex,” “being not respected,” “job-requirement is more than my ability,” “lack of stability at home,” “unclear promotion requirements,” and “no participation in department's decision making” with respective mean scores of 4.00, 3.86, 3.86, 3.71, 3.71, and 3.71. On the other hand, in the study in Germany and Austria, “against the conviction patients were kept alive by all means” (mean score 2.88), “stress due to patient's disease progression” (mean score 2.79), “high physical workload” (mean score 2.76), and “patients suffering of my therapy” (mean score 2.74) were the main sources of radiographers' workplace stress [32]. The variation with the current study might be due to differences in setting and sample size.

The strength of this study is this is the first of its kind in Ethiopia that determines workplace stress and associated factors among different health professionals. However, similar to other studies, this study has limitations of self-reported data bias.

## 5. Conclusion

A majority of health professionals significantly experienced workplace stress. Workload and working in night shift were significantly associated with workplace stress. Health policy makers and hospital managers should identify sources of workplace stress and should be concerned about workplace stress. Further large-scale study should be done in different parts of the country to provide strong evidence regarding the determinants of workplace stress among health professionals.

## Figures and Tables

**Table 1:** Sociodemographic variables and stress levels among health-care professionals in Felege-Hiwot Referral Hospital, Bahir Dar, Northwest, Ethiopia, 2015.

Variable	Overall *N*(%)	Stressed	
		Yes *N*(%)	No *N*(%)
Sex			
Male	109 (55.1)	74 (67.9)	35 (32.1)
Female	89 (44.9)	61 (68.5)	28 (31.5)
Age			
≤25	29 (14.6)	21 (72.4)	8 (27.6)
26–30	97 (49)	67 (69.1)	30 (30.9)
31–35	30 (15.2)	15 (50.0)	15 (50.0)
36–40	21 (10.6)	17 (81.0)	4 (19.0)
41–45	11 (5.6)	9 (81.8)	2 (18.2)
≥46	10 (5.1)	6 (60.0)	4 (40.0)
Marital status			
Married	111 (56.1)	76 (68.5)	35 (31.5)
Divorced	5 (2.5)	4 (80.0)	1 (20.0)
Single	82 (41.4)	55 (67.1)	27 (32.9)
Job			
Physician	45 (22.7)	15 (33.3)	30 (66.7)
Nurse/midwifery/anaesthetist/radiographer	114 (57.6)	93 (81.6)	21 (18.4)
Others	39 (19.7)	27 (69.2)	12 (30.8)
Educational level			
Diploma	55 (27.8)	49 (89.1)	6 (10.9)
Bachelor's degree	89 (44.9)	67 (75.3)	22 (24.7)
Doctorate degree	39 (19.7)	11 (28.2)	28 (71.8)
Master's degree	11 (5.6)	6 (54.5)	5 (45.5)
Postdoctoral degree	4 (2)	2 (50.0)	2 (50.0)
Religion			
Orthodox tewahedo	171 (86.4)	117 (68.4)	54 (31.6)
Muslim	15 (7.6)	9 (60.0)	6 (40.0)
Protestant	12 (6.1)	9 (75.0)	3 (25.0)

*Working unit*			
Outpatient department			
Surgical OPD	28 (14.1)	16 (57.1)	12 (42.9)
Psychiatrics OPD	3 (1.5)	1 (33.3)	2 (66.7)
Obstetrics and gynecology OPD	12 (6.1)	7 (58.3)	5 (41.7)
Pediatrics OPD	7 (3.5)	4 (57.1)	3 (42.9)
Accident and casualty department	7 (3.5)	5 (71.4)	2 (28.6)
Medical OPD	37 (18.7)	23 (62.2)	14 (37.8)
Inpatient department			
Surgical ward	30 (15.2)	19 (63.3)	11 (36.7)
Medical ward	21 (10.6)	12 (57.1)	9 (42.9)
OR	17 (8.6)	11 (64.7)	6 (35.3)
Psychiatry ward	2 (1)	1 (50.0)	1 (50.0)
Obstetrics and gynecology ward	25 (12.6)	17 (68.0)	8 (32.0)
Pediatrics ward	6 (3)	5 (83.3)	1 (16.7)
ICU	5 (2.5)	3 (60.0)	2 (40.0)
Other	6 (3)	4 (66.7)	2 (33.3)
Work experience			
1–7 years	117 (59.1)	79 (67.5)	38 (32.5)
8–14 years	53 (26.8)	34 (64.2)	19 (35.8)
15–21 years	14 (7.1)	13 (92.9)	1 (7.1)
22–28 years	9 (4.5)	6 (66.7)	3 (33.3)
28–35 years	5 (2.5)	3 (60.0)	2 (40.0)
Monthly income			
<5000 birr	137 (69.2)	121 (88.3)	16 (11.7)
5000–10000 birr	54 (27.3)	12 (22.2)	42 (77.8)
>10000 birr	7 (3.5)	2 (28.6)	5 (71.4)

**Table 2:** Job characteristics and level of workplace stress among health-care professionals in Felege-Hiwot Referral Hospital, Bahir Dar, Northwest Ethiopia, 2015.

Job characteristics	Overall *N*(%)	Stressed	
		Yes *N*(%)	No *N*(%)
Workload			
40 hr/week	55 (27.8)	28 (50.9)	27 (49.1)
41–50 hr/week	58 (29.3)	51 (87.9)	7 (12.1)
>50 hr/week	85 (42.9)	56 (65.9)	29 (34.1)
Working in night shift			
All the time	14 (7.1)	10 (71.4)	4 (28.6)
Sometimes	157 (79.3)	114 (72.6)	43 (27.4)
Not at all	27 (13.6)	11 (40.7)	16 (59.3)
Working on weekends			
All the time	26 (13.1)	20 (76.9)	6 (23.1)
Sometimes	149 (75.3)	105 (70.5)	44 (29.5)
Not at all	23 (11.6)	10 (43.5)	13 (56.5)
Working on night/weekend call duties in addition to daily work			
All the time	13 (6.6)	10 (76.9)	3 (23.1)
Sometimes	108 (54.5)	74 (68.5)	34 (31.5)
Not at all	77 (38.9)	51 (66.2)	26 (33.8)
Getting free time compensation			
All the time	8 (4.0)	5 (62.5)	3 (37.5)
Sometimes	96 (48.5)	70 (72.9)	26 (27.1)
Not at all	94 (47.5)	60 (63.8)	34 (36.2)
Demand conflict on time			
All the time	27 (13.6)	21 (77.8)	6 (22.2)
Sometimes	120 (60.6)	84 (70.0)	36 (30.0)
Not at all	51 (25.8)	30 (58.8)	21 (41.2)
Workplace offer support for stress relief			
All the time	13 (6.6)	12 (92.3)	1 (7.7)
Sometimes	48 (24.2)	33 (68.8)	15 (31.2)
Not at all	137 (69.2)	90 (65.7)	47 (34.3)
Believe that there is inadequate staffing to do the job properly			
Yes	156 (78.8)	108 (69.2)	48 (30.8)
No	42 (21.2)	27 (64.3)	15 (35.7)
Spend enough time with family			
No	164 (82.2)	119 (72.6)	45 (27.4)
Yes	34 (17.2)	16 (47.1)	18 (52.9)
Having close friends/family in workplace			
No	73 (36.9)	53 (72.6)	20 (27.4)
Yes	125 (63.1)	82 (65.6)	43 (34.4)
Workplace provide extracurricular activities that would decreased stress level			
No	152 (76.8)	101 (66.4)	51 (33.6)
Yes	46 (23.2)	34 (73.9)	12 (26.1)
Know whom to approach when stressed			
No	94 (47.5)	69 (73.4)	25 (26.6)
Yes	104 (52.5)	66 (63.5)	38 (36.5)

**Table 3:** Results of logistic analysis between dependent and independent variables among health professionals in Felege-Hiwot Referral Hospital, Bahir Dar, Northwest Ethiopia, 2015.

Variable	Stressed		95% CI		*p* value
	Yes *N*(%)	No *N*(%)	COR	AOR	
*Workload*					
>50 hr/week	56 (65.9)	29 (34.1)	1.86 (0.93–3.72)	9.63 (2.41–38.46)	0.001
41–50 hr/week	51 (87.9)	7 (12.1)	7.03 (2.71–18.17)	5.98 (9.93–35.99)	0.008
40 hr/week	28 (50.9)	27 (49.1)	1	1	

*Working in night shift*					
All the time	10 (71.4)	4 (28.6)	3.64 (0.90–14.60)	6.65 (0.65–67.73)	0.11
Sometimes	114 (72.6)	43 (27.4)	3.85 (1.65–8.96)	3.66 (1.00–13.28)	0.04
Not at all	11 (40.7)	16 (59.3)	1	1	

*Note*. COR: crude odds ratio, AOR: adjusted odds ratio, CI: confidence interval.

## Data Availability

The data used to support the findings of this study are available from the corresponding author upon request.

## Acronyms

AOR: Adjusted odds ratio
CCU: Coronary care unit
CI: Confidence interval
DEGRO: German Society of Radiation Oncology
FBAS: Stress questionnaire of physicians and nurses
HSE: Health safety executive
ICU: Intensive care unit
MOH: Ministry of Health
MS: Management standard
OR: Odds ratio
PSS: Perceived stress scale
SSO: Social security organization
UK: United Kingdom
WHO: World Health Organization.
